# Circadian clock–related transcription factors Reveille 1 and Reveille 8 regulate the *O*-acetylserine cluster genes and sulfur homeostasis

**DOI:** 10.1093/plphys/kiag372

**Published:** 2026-06-14

**Authors:** José María López Ramos, Karoline Snethlage, Hanna Bechtel, Anna Koprivova, Latifah Azeez, Suvajit Basu, Maximilian Klamke, Stanislav Kopriva

**Affiliations:** Institute for Plant Sciences, University of Cologne, Cologne Germany; Institute for Plant Sciences, University of Cologne, Cologne Germany; Institute for Plant Sciences, University of Cologne, Cologne Germany; Institute for Plant Sciences, University of Cologne, Cologne Germany; Institute for Plant Sciences, University of Cologne, Cologne Germany; Institute for Plant Sciences, University of Cologne, Cologne Germany; Institute for Plant Sciences, University of Cologne, Cologne Germany; Institute for Plant Sciences, University of Cologne, Cologne Germany

## Abstract

*O*-acetylserine (OAS) is the sulfur acceptor in primary sulfate assimilation and the precursor of cysteine, synthesized by serine acetyltransferase (SERAT). Beyond its metabolic role, OAS acts as a signaling molecule, accumulating during sulfur starvation and inducing sulfur-deficiency marker genes. Recently, OAS was also shown to increase transiently after a light–dark transition, triggering the induction of a group of “OAS cluster” genes. Here, we investigated the mechanisms underlying OAS accumulation and the regulation of OAS cluster genes. We found that no single SERAT isoform accounts for the full induction of OAS cluster genes; instead, the loss of any of the 3 major isoforms (SERAT1;1, SERAT2;1, SERAT2;2) reduced induction, with SERAT2;2 playing a predominant role. All 5 isoforms contributed to the response to varying degrees. In addition, 3 transcription factors were required: the sulfur-deficiency regulator SLIM1 and the circadian clock components RVE1 and RVE8. These factors bound to OAS cluster gene promoters and unexpectedly controlled OAS accumulation itself. While RVE1 and RVE8 did not transduce the OAS signal, they contributed to sulfur-deficiency responses at both the transcriptional and metabolic levels. Together, our findings reveal a complex regulatory network involving multiple SERAT isoforms and transcription factors, advancing our understanding of sulfur homeostasis and its integration with light and circadian regulation.

## Teaser

Transcription factors Reveille 1 and Reveille 8 contribute to regulation of the sulfate starvation response and are needed for induced accumulation of *O*-acetylserine after light–dark transition.

## Introduction

Sulfur is a vital nutrient for plant growth and development, with critical roles in numerous physiological processes. The main source of sulfur for plants is inorganic sulfate, which is taken up by roots and distributed through the plant by 4 groups of sulfate transporters (SULTR) (Takahashi et al. 2011). For assimilation into bioorganic molecules, sulfate is first activated by ATP sulfurylase, producing adenosine 5-phosphosulfate (APS) (Rotte and Leustek 2000). In the primary reductive sulfate assimilation pathway, APS is reduced to sulfite by APS reductase (APR), followed by reduction to sulfide by ferredoxin-dependent sulfite reductase (Takahashi et al. 2011). Sulfide is then incorporated into *O*-acetylserine (OAS) by *O*-acetylserine(thiol)-lyase (OAS-TL) to form cysteine (Maruyama-Nakashita 2017). Together with serine acetyltransferase (SERAT), which generates OAS from serine, OAS-TL forms the cysteine synthase complex (Wirtz et al. 2010). Cysteine biosynthesis occurs in plastids, mitochondria, and the cytosol, reflected by the presence of 3 major OAS-TL isoforms and 5 SERAT isoforms in *Arabidopsis* (Wirtz and Hell 2006). As the entry point for reduced sulfur, cysteine provides the building block for methionine, glutathione (GSH), and numerous coenzymes (Jobe et al. 2019).

Sulfate assimilation is tightly regulated by sulfur availability and metabolic demand (Takahashi et al. 2011). Under sulfur starvation, sulfate uptake and reduction are strongly upregulated. This response can also be triggered by OAS, light, carbohydrates, and reduced nitrogen compounds, while being repressed by abundant reduced sulfur or limited nitrogen and carbon (Koprivova and Kopriva 2014; Maruyama-Nakashita 2017). Phytohormones such as jasmonate, abscisic acid, salicylate, and cytokinins further modulate sulfur metabolism (Koprivova and Kopriva 2016). However, despite its importance for plants and animals, our understanding of sulfur sensing and signaling remains limited compared with other macronutrients such as nitrogen or phosphorus (Ristova and Kopriva 2022). Among the few known regulators, the transcription factor SULFUR LIMITATION 1 (SLIM1) is central to the sulfur deficiency response (Maruyama-Nakashita et al. 2006). SLIM1 mutants show reduced sulfate uptake, weaker transcriptional regulation of S-responsive genes, and impaired turnover of sulfur-containing secondary metabolites such as glucosinolates. SLIM1 belongs to the ETHYLENE-INSENSITIVE3-LIKE (EIL) family of transcription factors, which also includes EIL1, an additional regulator of sulfur deficiency that functions additively with SLIM1 (Dietzen et al. 2020).

Besides transcription factors, metabolites also act as important signaling molecules in sulfur metabolism. A key example is OAS, the direct precursor of cysteine. OAS accumulates under sulfur deficiency (Hirai et al. 2005; Hopkins et al. 2005), and exogenous OAS induces genes involved in sulfate assimilation (Neuenschwander et al. 1991). Genome-wide transcriptome analyses revealed that OAS treatment elicits a response that strongly overlaps with that of sulfur starvation (Hirai et al. 2003). Importantly, OAS directly induces a small group of genes known as the “OAS cluster,” identified through systems biology approaches combining metabolite profiles with transcriptomic datasets (Hubberten et al. 2012). The analysis used a correlation between increased accumulation of OAS after transition from light to dark and in the middle of the night with global transcriptomes at these conditions to identify candidate genes regulated by OAS (Espinoza et al. 2010; Caldana et al. 2011). The regulation by OAS was confirmed by transcriptome analysis of plants upon induction of OAS synthesis (Hubberten et al. 2012). The OAS cluster includes genes coding for Sulfur Deficiency Induced 1 (SDI1) and SDI2, 2 tetratricopeptide repeat proteins inhibiting synthesis of glucosinolates during sulfur deficiency (Aarabi et al. 2016); Response to Low Sulfur 1 (LSU1), one of 4 isoforms of small proteins that act as hubs in protein networks of stress responses (Sirko et al. 2014); the APR3 isoform APS reductase, a key enzyme of sulfate assimilation (Gutierrez-Marcos et al. 1996; Setya et al. 1996); a γ-glutamyl-cyclotransferase 2;1 (GGCT2;1) involved in glutathione recycling and homeostasis (Paulose et al. 2013); and a nuclear serine hydroxymethyltransferase SHM7, also named More Sulfur Accumulation1 (MSA1), important for DNA methylation in response to sulfur starvation (Huang et al. 2016). All six of these genes are strongly upregulated by sulfur deficiency (Hirai et al. 2003; Maruyama-Nakashita et al. 2003; Nikiforova et al. 2003), and with the exception of *APR3*, they are under SLIM1 control (Dietzen et al. 2020).

In this study, we aimed to uncover the molecular mechanisms coordinating the regulation of OAS cluster genes. Specifically, we examined which SERAT isoforms contribute to OAS accumulation after light–dark transitions, and we identified transcription factors binding to the promoters of all 6 OAS cluster genes. Finally, we investigated the roles of 3 such transcription factors in regulating the OAS cluster response.

## Results

### Interplay of SERAT isoforms in regulation of OAS cluster by light–dark transition

To investigate how OAS cluster genes are regulated after a light–dark transition, we first asked which SERAT isoform is required for OAS accumulation and the subsequent induction of gene expression. We replicated the experimental design of Hubberten et al. (2012) and Caldana et al. (2011), using mutants in the 5 SERAT isoforms, and measured transcript levels of the 6 OAS cluster genes in shoots of 17-d-old plants, 40 min after the transition.

**Figure 1:**
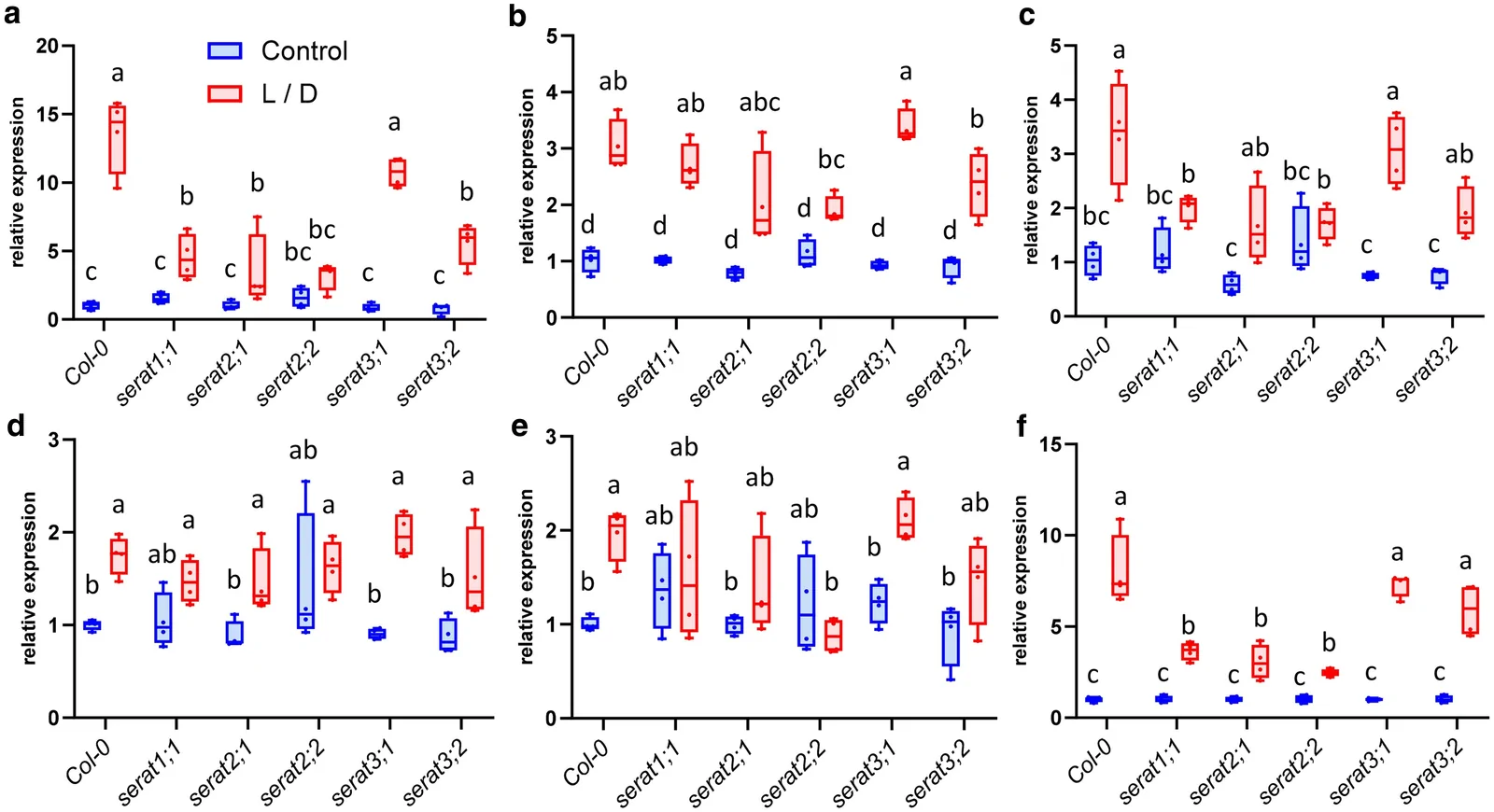
Regulation of OAS cluster genes by light-dark transition in *serat* mutants. *Arabidopsis* mutants in single isoforms of SERAT and the WT were grown for 17 d on agar plates and transferred to darkness. Two to 3 shoots were harvested and pooled as one sample 40 min after the transfer and from control plants grown in light for RNA isolation. Relative transcript levels of OAS cluster genes *SDI1* (a), *SDI2* (b), *LSU1* (c), *APR3* (d), *GGCT2;1* (e), and *SHM7* (f) were determined by RT-qPCR. The RT-qPCR results were obtained from 4 biological and 2 technical replicates; the transcript levels in WT Col-0 control were set to 1. In the box plots, each dot represents one biological replicate, n = 4; the central line indicates the median; the limits of the box show the upper and lower quartiles; and the whiskers indicate maximum and minimum values. Letters mark values significantly different at *P* < 0.05 (2-way ANOVA).

As expected, all OAS cluster genes were induced in wild-type (WT) plants (Fig. 1), with *SDI1* and *SHM7* showing the strongest responses. While the pattern of regulation of the 6 genes in the individual mutants varied, the loss of SERAT3;1 or SERAT3;2 had no effect on gene induction compared to WT, indicating that these cytosolic isoforms do not contribute to OAS synthesis during the light–dark transition. In contrast, *serat1;1*, *serat2;1*, and *serat2;2* mutants showed little or no induction for most genes (Fig. 1), suggesting that all 3 isoforms participate in the regulatory response. The *serat2;2* mutant, lacking the mitochondrial isoform, was most strongly affected, consistent with the importance of mitochondrial OAS synthesis for cysteine production (Watanabe et al. 2008).

**Figure 2:**
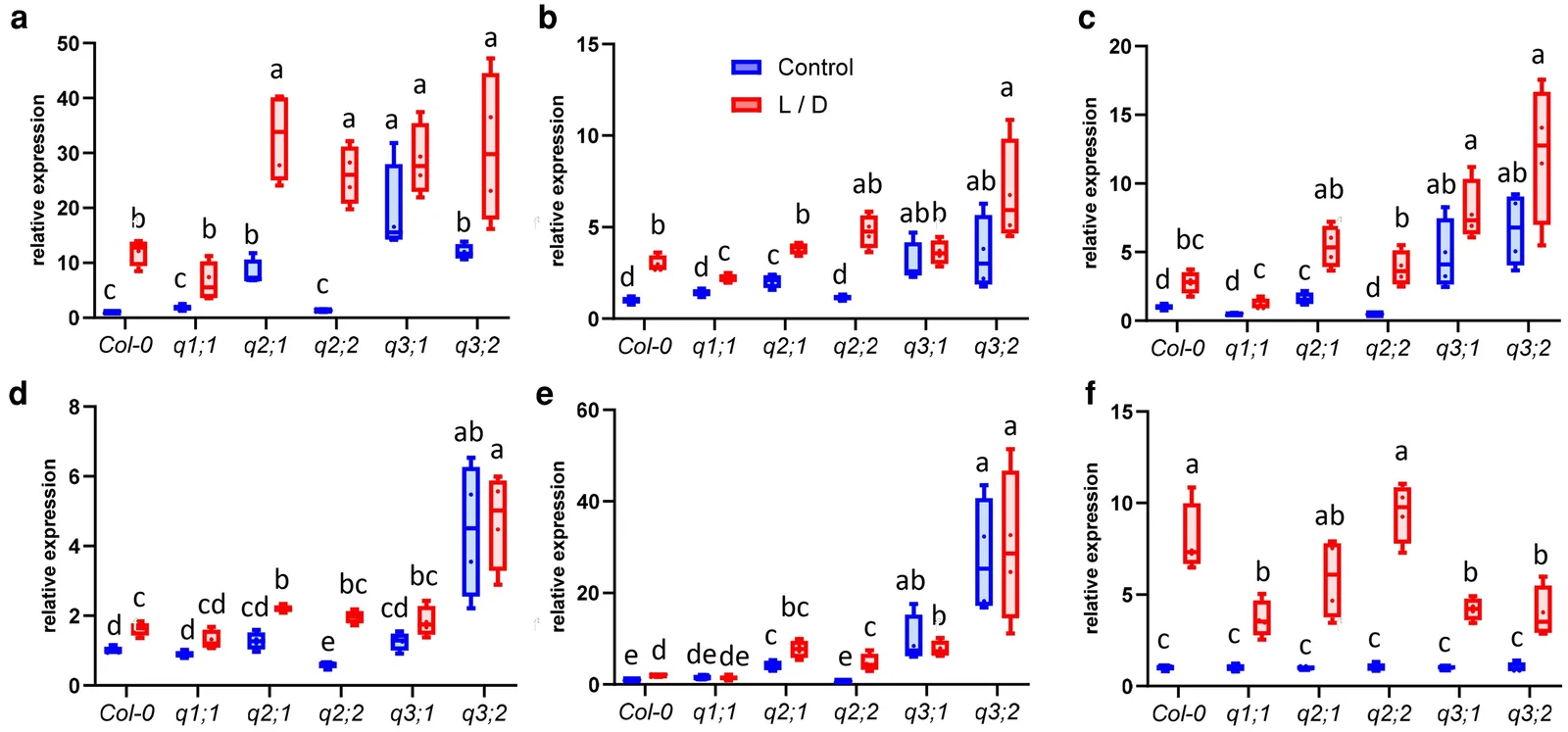
Regulation of OAS cluster genes by light–dark transition in quadruple *serat* mutants. *Arabidopsis* mutants with only one functional isoform of SERAT and the WT were grown for 17 d on agar plates and transferred to darkness. Two to 4 shoots were harvested and pooled as one sample 40 min after the transfer and from control plants grown in light for RNA isolation. Relative transcript levels of OAS cluster genes *SDI1* (a), *SDI2* (b), *LSU1* (c), *APR3* (d), *GGCT2;1* (e), and *SHM7* (f) were determined by RT-qPCR. The RT-qPCR results were obtained from 4 biological and 2 technical replicates; the transcript levels in WT Col-0 control were set to 1. In the box plots, each dot represents one biological replicate, n = 4; the central line indicates the median; the limits of the box show the upper and lower quartiles; and the whiskers indicate maximum and minimum values. Letters mark values significantly different at *P* < 0.05 (2-way ANOVA).

We next analyzed quadruple SERAT mutants, in which only a single isoform remains functional (Watanabe et al. 2008). Interestingly, several OAS cluster genes (except *SHM7*) showed elevated basal expression in *q3;2* (SERAT3;2 only), and in *q2;1* and *q3;1* four genes were upregulated (Fig. 2), consistent with decreased sulfate content in these mutants (Watanabe et al. 2018). However, the response of the quadruple mutants to light–dark transition differed. *SHM7* was consistently upregulated across all genotypes (Fig. 2). In mutants with only the minor cytosolic isoforms (*q3;1* and *q3;2*), OAS cluster genes were generally not induced, except for SDI1 in *q3;2*. On the other hand, the *q2;2,* containing only the mitochondrial isoform, displayed a response similar to WT (Fig. 2).

Together, these findings demonstrate that no single SERAT isoform fully accounts for the regulation of OAS cluster genes after light–dark transition. However, SERAT2;2 plays a central role, while SERAT1;1 and SERAT2;1 provide additional contributions.

### Transcription factors regulating the OAS cluster genes after light-dark transition

To identify transcription factors involved in regulating the OAS cluster genes, we used the Plant Regulomics platform, which integrates available ChIP-seq datasets (Ran et al. 2020). Seven transcription factors were predicted to bind to the promoters of all 6 OAS cluster genes: SLIM1, Reveille1 (RVE1), RVE8, Pseudo-response regulator PRR5, Leafy (LFY), the cysteine-rich polycomb-like protein (CPP) transcription factor SOL1, and WRKY33. From these, we selected SLIM1, RVE1, and RVE8 for further analysis, based on their roles in sulfur metabolism and circadian regulation. SLIM1 is the central regulator of sulfur deficiency responses (Maruyama-Nakashita et al. 2006). RVE8 is a core component of the circadian clock (Rawat et al. 2011), whereas RVE1 acts downstream of the clock and has also been implicated in auxin metabolism (Rawat et al. 2009).

**Figure 3:**
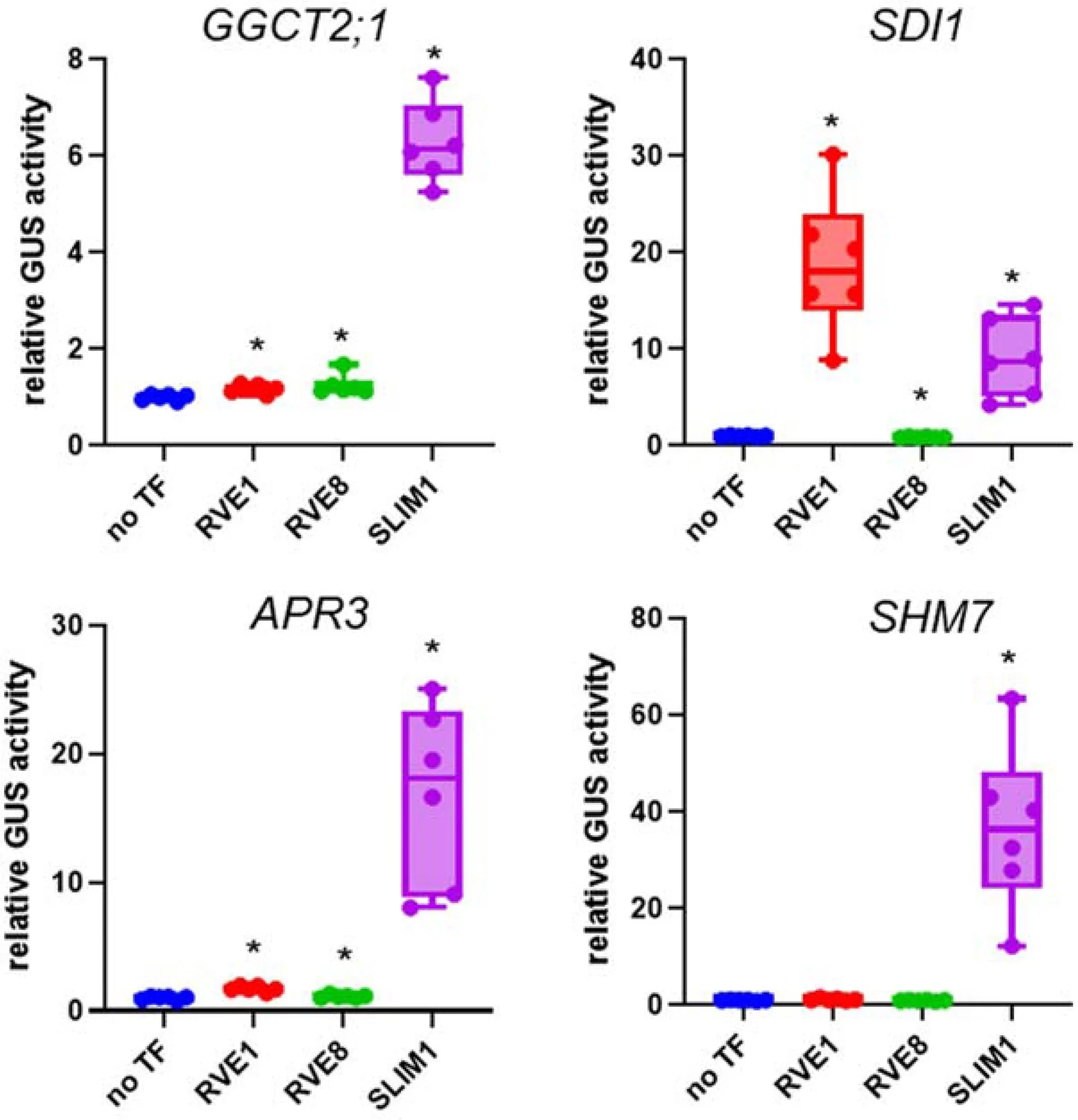
Quantification of the transactivation assays for interactions of RVE1, RVE8, and SLIM1 with promoters of OAS cluster genes. The promoters of *GGCT2;1, SDI1*, *APR3*, and *SHM7* genes were fused to the *uidA* (GUS) reporter gene. Cultured *A. thaliana* cells were transformed with the supervirulent *Agrobacterium* strain LBA4404.pBBR1MCS. virGN54D containing the reporter constructs and the effector constructs expressing RVE1, RVE8, and SLIM1 under control of 35S promoter. GUS activity was measured 3 d after the transformations. Promoter constructs without effectors were used as controls, and their activity was set to 1. In the box plots, each dot represents one biological replicate, n = 6; the central line indicates the median; the limits of the box show the upper and lower quartiles; and the whiskers indicate maximum and minimum values. Asterisks mark values significantly different from the no TF controls (*P* < 0.05, *t*-test).

We first tested the activity of these transcription factors towards OAS cluster promoters using transactivation assays in *Arabidopsis* cells (Berger et al. 2007). Cells were co-transformed with *Agrobacterium tumefaciens* carrying either promoter::GUS constructs or overexpression constructs of the transcription factors under the 35S promoter. GUS staining and measurement of GUS activity confirmed activity of all 3 factors towards the promoters of *SDI1*, *APR3*, and *GGCT2;1* (Fig. 3, Figure S1). While most interactions resulted in induction of GUS activity compared to controls without the effectors, RVE8 inhibited the activity controlled by *SDI1* promoter. On the other hand, only SLIM1 showed activity with *SHM7* promoter. SLIM1 showed the strongest activation of all promoters except *SDI1*, which was more strongly activated by RVE1. Together, these assays confirmed that SLIM1, RVE1, and RVE8 directly bind to and activate most OAS cluster gene promoters.

**Figure 4:**
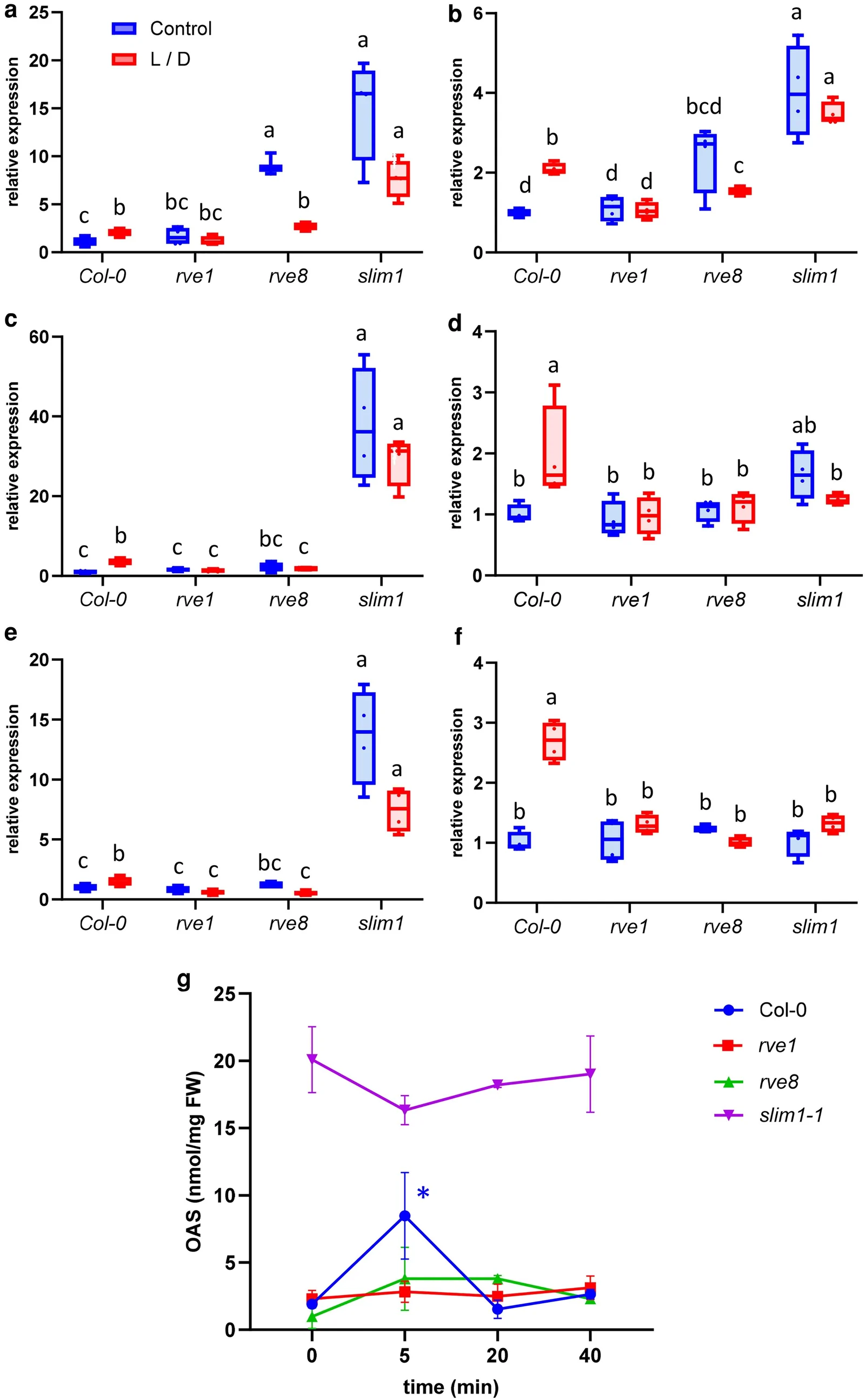
Regulation of OAS cluster genes and OAS accumulation by light–dark transition in mutants of transcription factors. *Arabidopsis rve1*, *rve8*, and *slim1-1* mutants and the WT were grown for 17 d on agar plates and transferred to darkness. Two to 3 shoots were harvested and pooled as one sample 40 min after the transfer and from control plants grown in light for RNA isolation. Relative transcript levels of OAS cluster genes *SDI1* (a), *SDI2* (b), *LSU1* (c), *APR3* (d), *GGCT2;1* (e), and *SHM7* (f) were determined by RT-qPCR. The RT-qPCR results were obtained from 4 biological and 2 technical replicates; the transcript levels in WT Col-0 control were set to 1. In the box plots, each dot represents one biological replicate, n = 4; the central line indicates the median; the limits of the box show the upper and lower quartiles; and the whiskers indicate maximum and minimum values. Letters mark values significantly different at *P* < 0.05 (2-way ANOVA). g) OAS was determined in shoots just before transition (0 min) and after 5, 20, and 40 min. Data from 4 biological replicates are shown as means and standard deviation. Asterisks mark values significantly different from T = 0 in the corresponding genotype (*t*-test).

Thus, to test their role *in vivo*, we analyzed homozygous mutant lines *slim1-1*, *rve1*, and *rve8* after light–dark transition. In contrast to WT plants, which showed induction of all OAS cluster genes 40 min after transition, none of the 6 genes were induced in the mutants (Fig. 4). As reported previously (Dietzen et al. 2020), basal transcript levels of *SDI1*, *SDI2*, *LSU1*, and *GGCT2;1* were already higher in *slim1-1* compared to WT. These results demonstrate that SLIM1, RVE1, and RVE8 are essential for induction of OAS cluster genes following the light–dark transition.

We next examined whether these transcription factors act upstream or downstream of OAS accumulation. OAS levels in shoots were quantified by HPLC after the transition (Fig. 4g). In WT plants, a transient OAS peak was observed after 5 min, whereas OAS concentrations remained stable in all 3 mutants. Notably, *slim1-1* plants maintained ∼4-fold higher OAS concentrations than WT across all time points. These results suggest that SLIM1, RVE1, and RVE8 not only regulate OAS cluster genes directly but also influence OAS metabolism upstream of its transient accumulation. However, it remains unclear whether they are required for transmitting the OAS signal itself.

We then tested whether the absence of the OAS peak in the mutants could be explained by altered regulation of *SERAT* genes. Previous work showed that the 3 main isoforms (*SERAT1;1*, *SERAT2;1*, and *SERAT2;2*), required for the transient OAS accumulation, are not under SLIM1 control (Dietzen et al. 2020). Chip-seq data showed RVE1 and RVE8 binding to these 3 SERAT promoters (Chow et al. 2019); however, expression analysis in *rve1* and *rve8* revealed that while the basal levels of *SERAT1;1* were slightly lower in the mutants than in the WT, the *SERAT2;1* and *SERAT2;2* were unaffected (Figure S2). Moreover, *SERAT1;1* expression was repressed by light–dark transition in WT but not in the *rve* mutants, while *SERAT2;1* and *SERAT2;2* remained unchanged in all genotypes. Thus, regulation of *SERAT* cannot explain the lack of OAS accumulation in *slim1-1*, *rve1*, and *rve8*.

Finally, we analyzed the expression of *RVE1*, *RVE8*, and *SLIM1* in the corresponding mutants. In WT plants, both *RVE1* and *RVE8* were downregulated 40 min after the light–dark transition (Figure S3). Loss of RVE1 led to a 2-fold increase in *RVE8* transcript levels under control conditions, whereas loss of RVE8 resulted in a 2-fold decrease in *RVE1* expression. *SLIM1* expression also decreased slightly but significantly after transition in WT plants, but not in the mutants. Interestingly, *slim1-1* mutation did not alter the basal levels or light–dark response of *RVE1* or *RVE8*.

Taken together, these results reveal that SLIM1, RVE1, and RVE8 are key regulators of OAS cluster genes. They act both directly, by binding and activating the promoters, and indirectly, by influencing OAS accumulation. The interplay between these transcription factors suggests a complex regulatory network that integrates sulfur metabolism with circadian and metabolic signals.

### SLIM1, RVE1, and RVE8 are not general parts of OAS signal transduction

**Figure 5:**
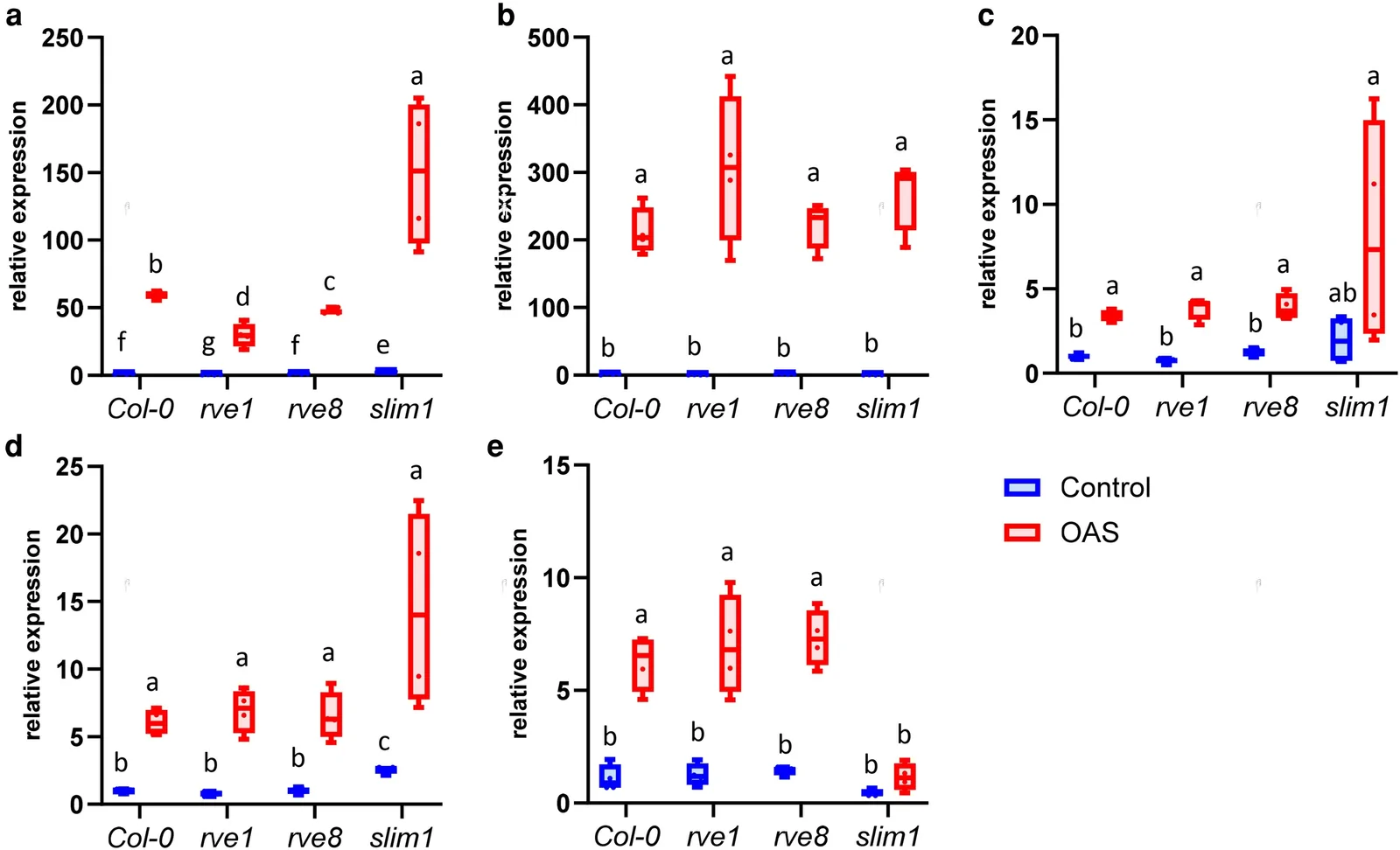
Regulation of OAS cluster genes by OAS in mutants of transcription factors. *Arabidopsis rve1*, *rve8*, and *slim1-1* mutants and the WT were grown for 14 d in hydroculture and treated with 1 mM OAS for 4 h. RNA was isolated from roots, and relative transcript levels of OAS cluster genes *SDI1* (a), *LSU1* (b), *APR3* (c), *SHM7* (d), as well as of sulfate transporter *SULTR1;2* (e), were determined by RT-qPCR. The RT-qPCR results were obtained from 4 biological and 2 technical replicates; the transcript levels in WT Col-0 control were set to 1. In the box plots, each dot represents one biological replicate, n = 4; the central line indicates the median; the limits of the box show the upper and lower quartiles; and the whiskers indicate maximum and minimum values. Letters mark values significantly different at *P* < 0.05 (2-way ANOVA).

Since the light–dark transition experiments suggested that SLIM1, RVE1, and RVE8 act upstream of OAS in the regulation of OAS cluster genes, we next asked whether these transcription factors are also involved in downstream OAS signaling. To test this, WT, *rve1*, *rve8*, and *slim1-1* plants were grown hydroponically for 2 weeks and then treated with 1 mM OAS for 4 h. Gene expression was analyzed in the roots, focusing on 4 OAS cluster genes (Fig. 5). In addition, we examined *SULTR1;2*, which encodes the high-affinity sulfate transporter essential for root sulfate uptake. *SULTR1;2* is known to be induced by OAS and regulated by SLIM1 (Maruyama-Nakashita et al. 2006). Reverse transcription quantitative PCR (RT-qPCR) analysis revealed that all 5 genes were induced by OAS in WT roots, with *LSU1* and *SDI1* showing the strongest upregulation (Fig. 5). Loss of RVE1 or RVE8 did not affect the induction of any of the genes, clearly indicating that these transcription factors are not part of the OAS signaling pathway. In contrast, the loss of SLIM1 function had gene-specific effects. While *SDI1*, *LSU1*, and *SHM7* were induced to a similar extent as in WT (notably, *SDI1* and *SHM7* were already elevated in untreated *slim1-1* roots), *APR3* and *SULTR1;2* failed to respond to OAS in the *slim1-1* mutant (Fig. 5). Finally, we examined transcription factor expression in the roots following OAS treatment. *RVE1* and *SLIM1* transcript levels were unaffected, while *RVE8* was significantly downregulated (Figure S4). Together, these results show that although SLIM1, RVE1, and RVE8 bind to the promoters of OAS cluster genes, only SLIM1 plays a role in their regulation by OAS, and its effects are gene specific.

### Loss of function of RVE1 and RVE8 impacts the response to sulfur deficiency

The connection between OAS and sulfur metabolism, along with SLIM1 as a key transcription factor in sulfur deficiency response, is well established. Our results showed that the transcription factors RVE1 and RVE8 also regulate OAS cluster genes, sometimes in a manner similar to SLIM1. We therefore hypothesized that RVE1 and RVE8 might participate in the sulfur deficiency response alongside SLIM1, analogous to the additive effect of EIL1 (Dietzen et al. 2020). To test this, *rve1*, *rve8*, and *slim1-1* mutants were subjected to sulfur deficiency for 17 days on agarose plates, and sulfur-containing metabolites and S-starvation marker gene expression were analyzed.

**Figure 6:**
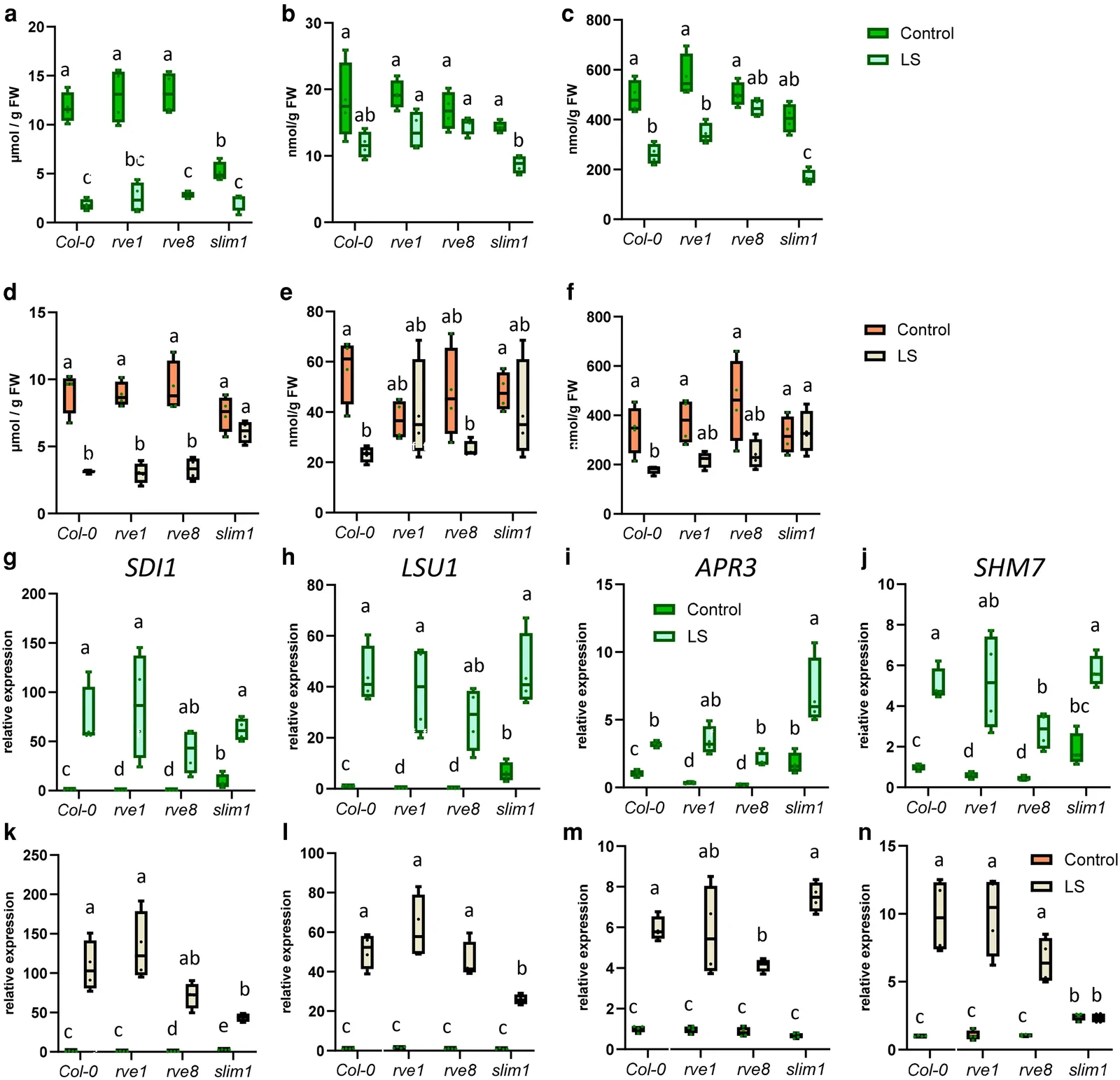
Involvement of RVE1, RVE8, and SLIM1 in sulfur starvation response. *Arabidopsis rve1*, *rve8*, and *slim1-1* mutants and the WT were grown for 17 d on agarose plates with control sulfate supply (750 µM) or sulfur limitation (LS, 25 µM sulfate). Two to 3 shoots and 4 to 5 roots were harvested and pooled as one sample. Sulfate was determined in the shoots (a) and roots (d) by ion chromatography. Cysteine (b, e) and glutathione (c, f) were measured in shoots (b, c) and roots (e, f) by HPLC. RNA was isolated from shoots (g-j) and roots (k-n), and relative transcript levels of OAS cluster genes *SDI1* (g, k), *LSU1* (h, l), *APR3* (i, m), and *SHM7* (j, n) were determined by RT-qPCR. The RT-qPCR results were obtained from 4 biological and 2 technical replicates; the transcript levels in WT Col-0 control were set to 1. In the box plots, each dot represents one biological replicate, n = 4; the central line indicates the median; the limits of the box show the upper and lower quartiles; and the whiskers indicate maximum and minimum values. Letters mark values significantly different at *P* < 0.05 (2-way ANOVA).

In both control and low S conditions, *slim1-1* plants were slightly smaller with shorter roots than the WT as well as *rve1* and *rve8* mutants (Figure S5). Sulfate concentrations were reduced in both shoots and roots under sulfur-deficient conditions, as expected (Fig. 6a, d). Under control conditions, *slim1-1* showed significantly lower foliar sulfate than WT, while sulfate levels in shoots under sulfur deficiency were comparable across all genotypes. In roots, loss of RVE1 and RVE8 had no effect on sulfate concentrations under either condition, whereas *slim1-1* retained sulfate, which did not decrease under S limitation (Fig. 6d). These results confirm that SLIM1 negatively affects sulfate allocation to the shoots (Maruyama-Nakashita et al. 2006; Dietzen et al. 2020), while RVE1 and RVE8 do not. Transcript levels of *SULTR2;1*, responsible for root-to-shoot transport, were increased by S-deficiency to a similar extent in the roots of all genotypes (Figure S6). In the shoots, *SULTR2;1* was not affected in *rve1* and *rve8*, however, it was upregulated in *slim1-1*, consistent with previous reports (Kawashima et al. 2009; Maruyama-Nakashita et al. 2015). Foliar cysteine levels were similar across genotypes under control and sulfur-deficient conditions, except for lower cysteine in *slim1-1* shoots (Fig. 6b). In roots, cysteine decreased under S deficiency in WT but remained stable in all mutants (Fig. 6e). Shoot glutathione levels were comparable under control conditions and decreased in all genotypes except *rve8* under S limitation (Fig. 6c). In roots, glutathione was slightly diminished by S deficiency in WT but unaffected in the mutants (Fig. 6f). The metabolite changes were not underlain by corresponding alterations in transcript of sulfite reductase (SiR), key component of sulfate reduction pathway (Figure S6c,d). Thus, loss of RVE1, RVE8, or SLIM1 attenuates the S-deficiency-induced decline of thiols in roots.

Next, we examined the expression of 4 OAS cluster genes—*SDI1*, *LSU1*, *APR3*, and *SHM7*—under S-deficiency. Consistent with previous studies (Dietzen et al. 2020), *slim1-1* mutants exhibited elevated transcript levels of *SDI1*, *LSU1*, and *APR3* in shoots under control conditions (Fig. 6g-j). Conversely, *rve1* and *rve8* mutants showed lower transcript levels for all 4 genes. Under S-deficiency, although the steady-state transcript levels were comparable, the magnitude of upregulation differed markedly. For example, *SDI1* was upregulated 70-fold in WT shoots, only 7-fold in *slim1-1*, but 300-fold in *rve1* and 120-fold in *rve8* (Fig. 6g). Similarly, *LSU1* induction reached 44-fold in WT, 182-fold in *rve1*, 170-fold in *rve8*, and only 4.5-fold in *slim1-1* (Fig. 6h). *APR3* and *SHM7* showed similar patterns, except that *APR3* induction in *slim1-1* was comparable to WT, consistent with it not being under SLIM1 control (Maruyama-Nakashita et al. 2006; Dietzen et al. 2020). In roots, the effect of the SLIM1 mutation was more pronounced: all genes except *APR3* were significantly less upregulated under S-deficiency compared to WT (Fig. 6k-n). In contrast, loss of RVE1 or RVE8 had only minor effects on transcript levels and gene induction in roots. These results indicate that RVE1 and RVE8 act as modulators of the transcriptional response to S-deficiency in shoots, functioning predominantly as repressors.

## DISCUSSION

### Complex network of SERAT isoforms is responsible for the induction of OAS cluster genes by light–dark transition

Understanding the molecular mechanisms of sulfur signaling in plants lags behind that of other major inorganic nutrients, nitrogen and phosphorus (Ristova and Kopriva 2022). While several transcription factors regulating sulfur starvation responses and other sulfur-related traits have been identified (Maruyama-Nakashita et al. 2006; Hirai et al. 2007; Dietzen et al. 2020), relatively little is known about the sensing mechanisms and signal transduction pathways. One important signaling molecule is OAS, which regulates sulfate reduction and the transcript levels of many sulfur-related genes (Neuenschwander et al. 1991; Hirai et al. 2003; Hubberten et al. 2012; Apodiakou and Hoefgen 2023). OAS was initially considered to be a signal of sulfur starvation, as it accumulates under sulfur-limited conditions, and also to convey information about nitrogen status, due to its influence on sulfur metabolism in nitrogen-starved plants (Koprivova et al. 2000; Hirai et al. 2003). Interestingly, OAS levels also rise transiently during transitions from light to dark or in the middle of the night, accompanied by induction of a specific set of OAS-cluster genes (Hubberten et al. 2012). However, the physiological relevance and underlying mechanisms of this induction remain unclear. To address this knowledge gap, we asked how OAS levels increase upon light–dark transition and how the OAS signal is translated into enhanced gene expression. This includes identifying the transcription factor(s) activated by increased OAS and determining the enzyme responsible for its accumulation.

OAS is synthesized by SERAT, which in *Arabidopsis* is encoded by a family of 5 genes and localized to plastids, mitochondria, and the cytosol (Haas et al. 2008; Watanabe et al. 2008). At least one functional SERAT isoform is essential for plant survival, but any single isoform is sufficient for *Arabidopsis* to complete its life cycle (Watanabe et al. 2008). We used a collection of single and quadruple SERAT mutants to identify which isoform drives the OAS increase during light–dark transition. We expected that loss of the relevant isoform would substantially reduce induction of the OAS cluster genes. Indeed, we observed diminished induction—but surprisingly, this occurred not in just one mutant, but in all mutants in the 3 major isoforms: *serat1;1*, *serat2;1*, and *serat2;2* (Fig. 1). This indicates that OAS synthesis in all 3 compartments contributes to the transient OAS peak and subsequent gene induction. This finding was unexpected, as the mitochondrial isoform SERAT2;2 had previously been shown to contribute most to OAS synthesis (Haas et al. 2008). Consistent with this, the *serat2;2* single mutant was the most strongly affected in the light–dark transition experiments, with *SDI1* induction abolished, whereas *serat1;1* and *serat2;1* mutants showed only partial attenuation (Fig. 1a). Analysis of quadruple mutants confirmed that each isoform alone can trigger induction of some OAS cluster genes, though not all genes respond equally. Notably, the mutant retaining only SERAT2;2 closely resembled the WT. Taken together, these results indicate that the mechanism underlying OAS accumulation upon light–dark transition and the associated gene induction is more complex than anticipated, requiring the interplay of 3 SERAT isoforms across 3 compartments, with a major role played by mitochondrial SERAT2;2. Moreover, despite being classified as a single cluster, the 6 OAS cluster genes display differential sensitivity to the loss of individual SERAT isoforms.

### SLIM1, RVE1, and RVE8 are needed for induction of OAS cluster genes by light–dark transition

The 6 OAS cluster genes are co-expressed not only in response to endogenous OAS (Hubberten et al. 2012) but also to exogenous OAS (Fig. 5; Hirai et al. 2003), sulfur deficiency (Maruyama-Nakashita et al. 2003; Nikiforova et al. 2003; Dietzen et al. 2020), and oxidative stress (Ristova and Kopriva 2022). Their coordinated expression, thus, likely depends on a shared set of transcription factors. Indeed, promoter analysis identified 7 transcription factors predicted to bind all 6 genes, including SLIM1, the central regulator of sulfur starvation responses (Maruyama-Nakashita et al. 2006). This aligns with the observation that most OAS cluster genes are highly induced by sulfur deficiency and, except for *APR3*, depend on SLIM1 for this induction (Dietzen et al. 2020). Three transcription factors—RVE1, RVE8, and PRR5—are associated with the circadian clock. PRR5 represses the core clock genes *CCA1* and *LHY* as part of a feedback loop (Nakamichi et al. 2010), and both *RVE1* and *RVE8* are direct targets of PRR5. Sulfate assimilation is circadian regulated, affecting both gene transcription and metabolic fluxes through primary and secondary pathways (Kopriva et al. 1999; Huseby et al. 2013). The other transcription factors binding to promoters of OAS cluster genes have different functions. WRKY33 is a key part of plant immune responses (Birkenbihl et al. 2012), so given the importance of sulfur for plant immunity (Rausch and Wachter 2005; Wang et al. 2022), this association is not surprising. On the other hand, LFY and SOL1 are involved in development, representing a key factor for floral meristem identity and a regulator of cell fate and cell division in the stomatal lineage, respectively (Weigel et al. 1992; Simmons et al. 2019), though their links to regulation of OAS cluster genes remain unclear.

Using the original light–dark transfer experiment that identified the OAS cluster, we confirmed that SLIM1, RVE1, and RVE8 affect OAS cluster gene expression (Fig. 4). Transactivation assays verified their binding to *SDI1*, *APR3*, and *GGCT2;1* promoters (Fig. 3), consistent with ChIP-seq data. SLIM1 produced the most consistent strong activation, agreeing with its central role in sulfur starvation response. Interestingly, *APR3* is upregulated by sulfur deficiency independently of SLIM1 but can still be activated by SLIM1 in transactivation assays, suggesting alternative transcription factors also contribute to its expression.

Although SLIM1, RVE1, and RVE8 bind to OAS cluster promoters and are necessary for transcript induction during light–dark transition, their loss also abolishes the transient OAS peak. This indicates they act upstream of OAS accumulation. The mechanism is unclear, as transcript levels of *SERAT2;1* and *SERAT2;2*, necessary for OAS production, are unaffected in these mutants, and *SERAT1;1* is only slightly reduced in *rve1* and *rve8* (Figure S2). Also, the transcriptional regulation of the transcription factors themselves is not able to explain the observed lack of OAS peak (Figure S3). In *slim1-1*, higher basal OAS levels may mask the transient light–dark peak (Fig. 4g), possibly due to low sulfate content (Fig. 6a) and pre-activation of sulfur starvation responses in the mutant (Figs. 1 and 2, 4 and 6). It is possible that the increased OAS content in *slim1-1* is a consequence of this lower sulfate content; however, OAS also accumulates in response to other stresses that lead to accumulation of reactive oxygen species (Apodiakou and Hoefgen 2023). The roles of RVE1 and RVE8 in OAS production upon light–dark transition remain to be clarified.

### Transcriptional regulation by OAS is independent from RVE1 and RVE8

Since the 3 transcription factors bind to promoters of the OAS cluster genes, we expected that they would be necessary for their induction by OAS (Hirai et al. 2003; Hubberten et al. 2012). However, RVE1 and RVE8 were not required for gene induction by exogenous OAS (Fig. 5). Thus, their activity towards promoters of the OAS cluster genes is not part of OAS signaling. Nevertheless, *RVE8* is downregulated by OAS (Figure S4), suggesting a potential role in longer-term gene induction, likely through its function as a repressor (Fig. 4). SLIM1's function is more nuanced: while some genes respond normally to OAS in *slim1-1*, *APR3* and *SULTR1;2* do not. (Figure 5). The lack of involvement of SLIM1 in response to OAS is unexpected, as the transcriptional networks of OAS treatment and S-starvation largely overlap (Hirai et al. 2003), the 3 genes, *SDI1*, *LSU1*, and *SHM7*, are under control of SLIM1 in the S-deficiency response, and SLIM1 showed a transcriptional activity on the promoters of these genes (Fig. 3). This suggests that OAS signaling and sulfur starvation responses are partially uncoupled, reviving questions about whether OAS is the true sulfur deficiency signal (Hubberten et al. 2012; Apodiakou and Hoefgen 2023). The SLIM1-controlled S-deficiency responsive genes do not lose the response in *slim1-1* (or *eil3*) mutants; the response is only attenuated (Maruyama-Nakashita et al. 2006; Dietzen et al. 2020). The SLIM1-independent induction by OAS might then be responsible for this attenuated response. However, what is/are the transcription factor(s) responsible for gene induction upon OAS feeding and how S-deficiency and OAS signaling converge to regulate OAS cluster genes (and many more genes) are emerging open questions.

Interestingly, *APR3* differs strongly from the other OAS cluster genes. It has been long known that *APR3* and the other APR isoforms are not under control of SLIM1 for induction by S-deficiency; therefore, the finding that SLIM1 is necessary for its induction by OAS is unexpected (Fig. 5c). It is, though, consistent with SLIM1 binding to *APR3* promoter (Fig. 3). At the same time, since OAS response of APR3 is dependent on SLIM1 and the S-starvation response is not, this supports the conclusion that OAS does not play a direct role in S-starvation response. Also, *SULTR1;2* depends on SLIM1 for its induction by OAS (Fig. 5e), as well as by S-deficiency (Dietzen et al. 2020). Thus, for some genes SLIM1 controls only S-deficiency response; for *APR3*, only OAS-induction; and for *SULTR1;2*, both. Exogenous OAS did not affect the expression levels of *SLIM1*, in accordance with the lack of changes in its mRNA levels under sulfur deficiency (Maruyama-Nakashita et al. 2006). Thus, to the unknown molecular mechanisms of SLIM1 function in S-deficiency response, a new question emerged, how SLIM1 translates OAS signal to some but not all OAS-induced genes.

### RVE1 and RVE8 are members of a sulfur starvation regulatory network

The involvement of RVE1 and RVE8 in sulfur starvation (Fig. 6) adds 2 factors to the small list of confirmed regulators of this response. SLIM1 controls the majority of the response, but residual induction in *slim1-1, eil3*, or *eil1* mutants indicates additional factors exist (Dietzen et al. 2020). From differentially expressed genes after S-starvation, 83% were under SLIM1 control in the roots, but only 44% in the leaves (Dietzen et al. 2020). Indeed, while predicted transcriptional networks suggest over 50 potential regulators, few have been experimentally validated (Kasajima et al. 2007; Bielecka et al. 2014; Frerigmann and Gigolashvili 2014; Henriquez-Valencia et al. 2018; Canales et al. 2020; Rakpenthai et al. 2022; Apodiakou and Hoefgen 2023; Fernandez et al. 2024). SLIM1 is part of the ethylene-insensitive 3-like family; and, thus, it is not surprising that other factors of the family, EIL1, EIL2, and EIN3, are also part of the transcriptional response (Wawrzynska et al. 2010; Wawrzynska and Sirko 2016; Dietzen et al. 2020). Other transcription factors from bZIP, MYB, ERF, or bHLH families were predicted through co-expression and network analyses and/or exploitation of available ChIP-seq data, but without analyses of the response to S-deficiency of corresponding mutants. RVE8 was also predicted to be part of the network (Apodiakou and Hoefgen 2023), as was RVE2 but not RVE1 (Henriquez-Valencia et al. 2018; Fernandez et al. 2024). Our mutant analysis confirms that RVE1 and RVE8 regulate sulfur homeostasis, as in the shoots of *rve1* and *rve8*, the steady state transcript accumulation of key marker genes was lower than in the WT (Fig. 6). They also clearly contribute to sulfur deficiency regulation, primarily in the shoots, acting as repressors of the transcriptional induction (Fig. 6), possibly to prevent overaccumulation of the upregulated transcripts. Both RVE1 and RVE8 are part of the circadian clock and have a similar expression pattern with maxima at dawn, but belong to separate clades (Rawat et al. 2011). As the genes for sulfate assimilation undergo a circadian rhythm (Kopriva et al. 1999; Harmer et al. 2000; Espinoza et al. 2010), it could be speculated that in the *rve1* and *rve8* mutants, the phase of the clock is shifted and affects the expression pattern of OAS cluster genes. However, the mutations have a different impact on the clock; while loss of RVE8 leads to a longer period, in *rve1*, the period is not affected (Rawat et al. 2009; Rawat et al. 2011). Therefore, the molecular mechanism of the regulation must be different and possibly more direct.

The alterations in transcriptional response to S-deficiency were accompanied by changes in metabolite accumulation, which were surprisingly more pronounced in the roots than in the shoots. The changes in thiols, which were less affected by the S-deficiency in the mutants than in WT, are consistent with a higher degree of upregulation of the genes, and thus a more efficient response to the starvation (Fig. 6). On the other hand, in *slim1-1* the shoots are more affected by the S-deficiency, in agreement with the expression pattern and with previous studies (Maruyama-Nakashita et al. 2006; Dietzen et al. 2020). This might be explained by a lower S-deficiency–induced translocation of sulfate to the shoots in *slim1-1* mutant. While in the WT plants miR395 restricts *SULTR2;1* expression to the xylem to optimize the translocation, this is not the case for *slim1-1* mutant (Kawashima et al. 2009). Increased retention of sulfate in the roots of *slim1-1* may enhance its reduction through sulfate assimilation, the genes for key enzymes of which, APR and SiR, are not under control of SLIM1 (Fig. 6, S6), thus, maintaining stable levels of cysteine and GSH (Fig. 6). Loss of RVE1 and RVE8 similarly lowers the impact of S-deficiency on the thiol accumulation in the roots, but the mechanism is not as obvious as for *slim1-1*. So, while RVE1 and RVE8 clearly are involved in the regulation of S-deficiency response, their effects are likely distinct from SLIM1, fine-tuning gene expression rather than serving as primary activators. Whether any of the proposed transcription factors control the residual response to S-starvation next to SLIM1 and EIL1 is thus still an open question. It is, however, possible that in analogy with the regulation of glucosinolates synthesis (Chen et al. 2024), there is not one other major factor, but a large number of transcription factors fine-tuning the response in different temporal and spatial manner.

In conclusion, we showed that all 3 major SERAT isoforms are required for OAS cluster gene induction upon light–dark transition. SLIM1, RVE1, and RVE8 directly regulate these genes, acting upstream of OAS accumulation, though only SLIM1 is involved in translating OAS into transcriptional activation for some genes. RVE1 and RVE8 are newly identified transcription factors controlling sulfur starvation responses in shoots, acting as repressors. Overall, our results suggest that responses to sulfur starvation and to exogenous OAS are controlled by distinct, partially overlapping regulatory mechanisms.

## Materials and methods

### Plant material

*Arabidopsis thaliana* ecotype Col-0 was used as WT in this study, and all mutants were in the Col-0 genetic background. The mutant lines for SERAT isoforms, single and quadruple, were provided by Dr. Rainer Höfgen, Max Planck Institute for Molecular Plant Physiology Golm, and described previously in Watanabe et al., (2008). These include: *serat1;1* (AT5G56760, SALK_05021), *serat2;1* (AT1G55920, SALK_099019), *serat2;2* (AT3G13110, Kazusa_KG752), *serat3;1* (AT2G17640, SALK_030223), *serat3;2* (AT4G45640, SALK_030011), *q1;1*, *q2;1*, *q2;2*, *q3;1*, and *q3;2*. The *slim1-1* was obtained from Hideki Takahashi, Michigan State University, and described in Maruyama-Nakashita et al., (2006). The mutants in RVE1 (AT5G17300, SALK_025754) and RVE8 (AT3G09600, SALK_053482C) were obtained from NASC and described previously (Rawat et al. 2009; Rawat et al. 2011). For the transactivation experiments *Arabidopsis thaliana* Ler-0 cell culture was used (Berger et al. 2007).

### Growth conditions

Seeds were surface sterilized using chlorine gas under the fume hood for 3 h by adding 2.5 mL of concentrated 37% (V/V) HCl to 125 mL sodium hypochlorite in a desiccator. Seeds were then sown under sterile conditions either onto agarose plates or in 12-well hydroculture system. For the light–dark transitions, sterilized seeds were placed on modified Long Ashton Medium agarose plates containing sucrose (Table S1), stratified in darkness at 4 °C for 3 d, and grown for 5 d in Panasonic light chambers at 22 °C under long day conditions (16-h light/8-h darkness; 150 µE*m^−2^*s^−1^). Afterwards, they were transferred to new plates containing the same medium but without sucrose and kept for another 12 d. Shoot samples, corresponding to 2 to 4 whole rosettes pooled together, depending on the size of the plants, were taken 4 h after subjective dawn (Time 0), and plants were transferred immediately to darkness. With the help of a green lamp, shoot samples were taken in the dark 5, 20, and 40 min after the transition.

For sulfur starvation experiments, seeds were placed on plates containing the modified Long Ashton Medium with either full sulfate supply (750 µM, control), or low sulfur supply (25 µM, LS). After stratification for 3 d, the plates incubated for 17 d in Panasonic light chambers at the same conditions. Samples from shoots and roots were collected in 1.5-mL tubes from different plates and immediately frozen in liquid nitrogen, securing at least 4 biological replicates for each treatment. To compare plant phenotypes after long S starvation, plants were grown in pots with a mixture of 90% washed quartz sand and 10% soil in growth chambers at short days (10-h light/14-h dark), 22 °C, and light intensity of 150 µE*m^−2^*s^−1^. The plants were watered with the modified Long Ashton Medium with either full sulfate supply (750 µM, control), or low sulfur supply (25 µM, LS). After 5 weeks the plants were photographed.

Alternatively, for OAS treatment, 1 mL of liquid modified Long Ashton Medium was placed in every well of 12-well plates. Seeds were placed on a sterile square polypropylene mesh floating on the nutrient solution; the plates were wrapped in aluminum foil and stored in darkness at 4 °C for 3 d. They were then moved to a Percival growth chamber under long day conditions (as described before), and the aluminum foil was removed after 3 d. Plants were grown for 2 weeks, and the media was replaced after every week. On d 15, 1 mM OAS was added to the liquid media, and root samples were collected after 4 h as described before.

### Anion analysis

Anions were analyzed by ion chromatography (IC) as described in Dietzen et al., (2020). Frozen samples were homogenized by using a Bead Ruptor 24 3D (Omni International, USA), 1 mL of sterile water was added to each tube, and they were incubated at 4 °C and 1500 rpm for 1 h. Next, samples were kept at 95 °C for 15 min, vortexed, and centrifuged at maximum speed for 15 min. The supernatant was transferred to IC vials and diluted 1:5 with water. Anions were separated by using a Dionex ICS-1100 chromatography system with a Dionex IonPac AS22 RFIC 4x 250-mm analytic column (Thermo Scientific, Germany) and 4.5 mM NaCO_3_/1.4 mM NaHCO_3_ as running buffer. External standards of nitrate, phosphate, and sulfate (0.5, 1, and 2 mM mix of KNO_3_, KH_2_PO_4_, and K_2_SO_4_) were used to determine anion concentrations in the samples.

### Quantification of low-molecular-weight thiols

The thiols cysteine and GSH were determined by HPLC according to (Dietzen et al. 2020). Frozen samples were homogenized as described before, a 10-fold amount (V/w) of 0.1 M HCl was added to each tube, and they were vortexed vigorously. Samples were centrifuged at maximum speed at room temperature, and 60 µL supernatant was transferred to a new tube. One hundred microliters of 0.25 mM CHES-NaOH pH 9.4 was added to each tube, together with 35 µL of 10 mM DTT; the samples were vortexed, and incubated for 40 min at room temperature. Afterwards, 5 µL of 25 mM monobromobimane was added, and the extracts were again vortexed and incubated at room temperature for 15 min in the dark. The reaction was stopped with the addition of 110 µL of 100 mM methanesulfonic acid, and samples were vortexed and centrifuged at 4 °C for 20 min. The thiols were determined in the supernatant by HPLC (Dionex UltiMate 3000 system [Thermo Fisher Scientific]) using an LC column 250 × 4.6 mm (Spherisorb ODS2, 5 μm) and fluorometric detection (excitation: 390 nm, emission: 480 nm). Two eluents were used in a linear gradient from 95% to 82% A (10% methanol, 0.25% acetic acid, pH 4.1) in B (90% methanol, 0.25% acetic acid, pH 4.1) with a constant flow rate of 1 mL/min, and the thiols were quantified using standards.

### Determination of *O*-acetylserine

OAS was determined using a pre-column derivatization with *O*-phtalaldehyde (Krueger et al. 2009). Frozen samples were homogenized as described before and extracted with 400 µL of 80% ethanol in 2.5 mM HEPES pH 6.8, shaken at 2000 rpm for 20 min at room temperature, and centrifuged at 4 °C for 12 min at maximum speed. Supernatants were collected in new tubes, and pellets were re-extracted with 400 µL of 50% ethanol in 2.5 mM HEPES pH 6.8, and once again with 200 µL of 80% ethanol in 2.5 mM HEPES pH 6.8. After centrifuging the combined supernatants for 5 min at room temperature at maximum speed, the OAS was determined by HPLC (Dionex UltiMate 300 system [Thermo Fisher Scientific]). The samples were derivatized online with *O*-phtalaldehyde (Dr. Maisch GmbH, Germany), loaded onto a reverse phase column (HyperClone, 3 mm, 150 × 4.6 mm ODS [C18], 120 A) and detected fluorometrically (emission: 330 nm; excitation: 450 nm). Two eluents, A (964 mL H_2_O, 33 mL 0.4 M NaPO_4_ [pH 6.8], 3 mL tetrahydrofuran) and B (225 mL H_2_O, 110 mL acetonitrile, 175 mL MeOH, 25 mL 0.4 M NaPO_4_ [pH 6.8]), were used for separation (0–2 min, 100% A; 2–21 min, 87% A, 13% B; 21–28.25 min, 85% A, 15% B; 28.25–37.3 min, 50% A, 50% B; 37.3–48.3 min 40% A; 48.3–63.3 min, 100% B; 63.3–65 min, 100% A) with a constant flow rate of 0.8 mL/min.

### Expression analysis

RNA was isolated by a standard phenol–chloroform extraction and LiCl precipitation. RNA concentration and purity was determined using NanoDrop 2000c Spectrophotometer (Thermo Scientific). For the RT-qPCR, 800 ng RNA was subjected to DNase treatment and reverse transcription using the QuantiTect Reverse Transcription Kit (Qiagen, Germany) according to the manufacturer's instruction. qPCR reactions (4 μL primer mix, 1 μL cDNA, and 5 μL GoTaq qPCR Master Mix [Promega]) were performed in a CFX96 Touch Real-Time PCR Detection System (Bio-Rad, Germany). Transcript levels were quantified using CFX Manager Software (Bio-Rad, Germany) and normalized to *TIP42 INTERACTING PROTEIN OF 41 KDA* (*TIP41*, AT4G34270) using the 2-ΔΔCT method. Primer sequences are listed in Table S2.

### Transactivation assays

For the transactivation assays, the procedure from Berger et al. (2007) was followed. The promoters of *SDI1*, *APR3*, *SHM7*, and *GGCT2;1* as well as genes encoding RVE1, RVE8, and SLIM1 were amplified from *Arabidopsis* genomic DNA using Phusion polymerase (New England Biolabs). After electrophoresis, the amplicons were purified using the ReliaPrep DNA Clean-up and Concentration System (Promega). Purified fragments were then ligated into pENTRY-D-TOPO vector using the TOPO Cloning Kit (Thermo Fisher Scientific). Plasmids were isolated using the PureYield Plasmid Miniprep System (Promega), and the inserts were verified by sequencing. The promoters were introduced into the destination vectors pGWB3 fusing them with the GUS reporter gene, while the transcription factors were integrated into pGWB2 to be expressed under control of 35S promoter using the Gateway LR Clonase Kit (Berger et al. 2007; Nakagawa et al. 2007). After confirmation via sequencing, plasmids were used to transform competent *Agrobacterium tumefaciens* LBA4404.pBBR1MCS virGIN 54D.

*A. tumefaciens* containing the constructs of interest, as well as the RK19 strain, were incubated overnight in liquid YEB medium with the respective antibiotics. Bacteria were pelleted, washed and resuspended in AT-medium (Table S1), which was also used to dilute the *Arabidopsis* cell culture 1:4. In 6-well plates, 4 mL of diluted cell suspension was pipetted, together with 20 µL of RK19 suspension. Then the different combinations of *Agrobacteria* with reporter and effector constructs were added. *A. tumefaciens* containing pGWB2_pro*35s::GUS* was used as a positive control, and a pGWB3 without any effector was used as negative control. Additionally, non-transformed cell culture was included as another negative control. Each combination was transformed thrice. Plates were covered in aluminum foil and shaken softly at room temperature in a growth chamber for 3 d. After incubation, 1 mL per well was harvested for quantitative analysis. Afterwards, 50 µL of X-Gluc (10 mg/mL; diluted in dimethylformamide) was added into each well; plates were covered again and incubated at 37 °C. Positive control wells turned blue after 2 h. and the other combinations were incubated overnight.

### Quantitative GUS assays

A crude extract from the 1-mL samples was prepared using 1 mL extraction buffer (50 mM NaPO4 pH 7.2, 10 mM EDTA, 1% Triton X-100). Total protein concentration was determined using the Pierce BCA Protein Assay Kit (Thermo Fisher Scientific) following the plate reader protocol combined with a BSA standard curve. GUS activity in extracts was determined with 4-methylumbelliferyl β-D-glucuronide (4-MUG) as a substrate by measuring 4-methyl umbelliferone (4-MU) fluorescence (excitation at 365 nm, emission at 455 nm) in black 96-well plates. To 25 μL of extract, 200 μL assay buffer (extraction buffer + 1.5 mM 4-MUG) was added, and fluorescence was measured in the Tecan Infinite 200 plate reader at 37 °C for 36 cycles in 5-min intervals. As a blank, 25 μL extraction buffer was measured, and each sample was measured in 3 biological replicates.

The mean fluorescence intensity was determined for each well at each time point after subtraction of blank. By using 4-MU standard curve, the amount of product at each time point was calculated. Promoter activity was determined from the slope of product amount as a function of time and protein concentration in the samples. Normalization was performed using the values of the promoter constructs without added effectors.

### Data processing and statistical analysis

Data was processed by using Microsoft Excel (Office 365; version 16.58) and GraphPad Prism (version 8.0) for visualization and generation of graphs. Four biological replicates were taken for every measurement, and the experiments were repeated at least twice. Statistical differences were calculated performing a 2-way ANOVA in RStudio when there were 2 independent variables and 1-way ANOVA/2-tailed, unpaired Student's *t*-test when there was only one.

### Declaration of AI-assisted technologies in the writing process

The authors used ChatGPT (OpenAI, San Francisco, California) to assist with language refinement and clarity improvements. All content was written, verified, and approved by the authors without reliance on AI for original scientific interpretation or conclusions.

### Accession numbers

Sequence data from this article can be found in the GenBank/EMBL data libraries under accession numbers NM_001343488.1, NM_001337828.1, and NM_106032.5.

## Supplementary Material

kiag372_Supplementary_Data

## Data Availability

The data underlying this article are available in the article and in its online supplementary material.
