# Supplementary material for: Circadian clock–related transcription factors Reveille 1 and Reveille 8 regulate the O-acetylserine cluster genes and sulfur homeostasis
Source: Plant Physiol. 2026 Jun 14;201(4):kiag372. doi: 10.1093/plphys/kiag372 (PMC13524097; doi:10.1093/plphys/kiag372)
Supplement: Supplementary file 1 [file kiag372_supplementary_data.pdf]

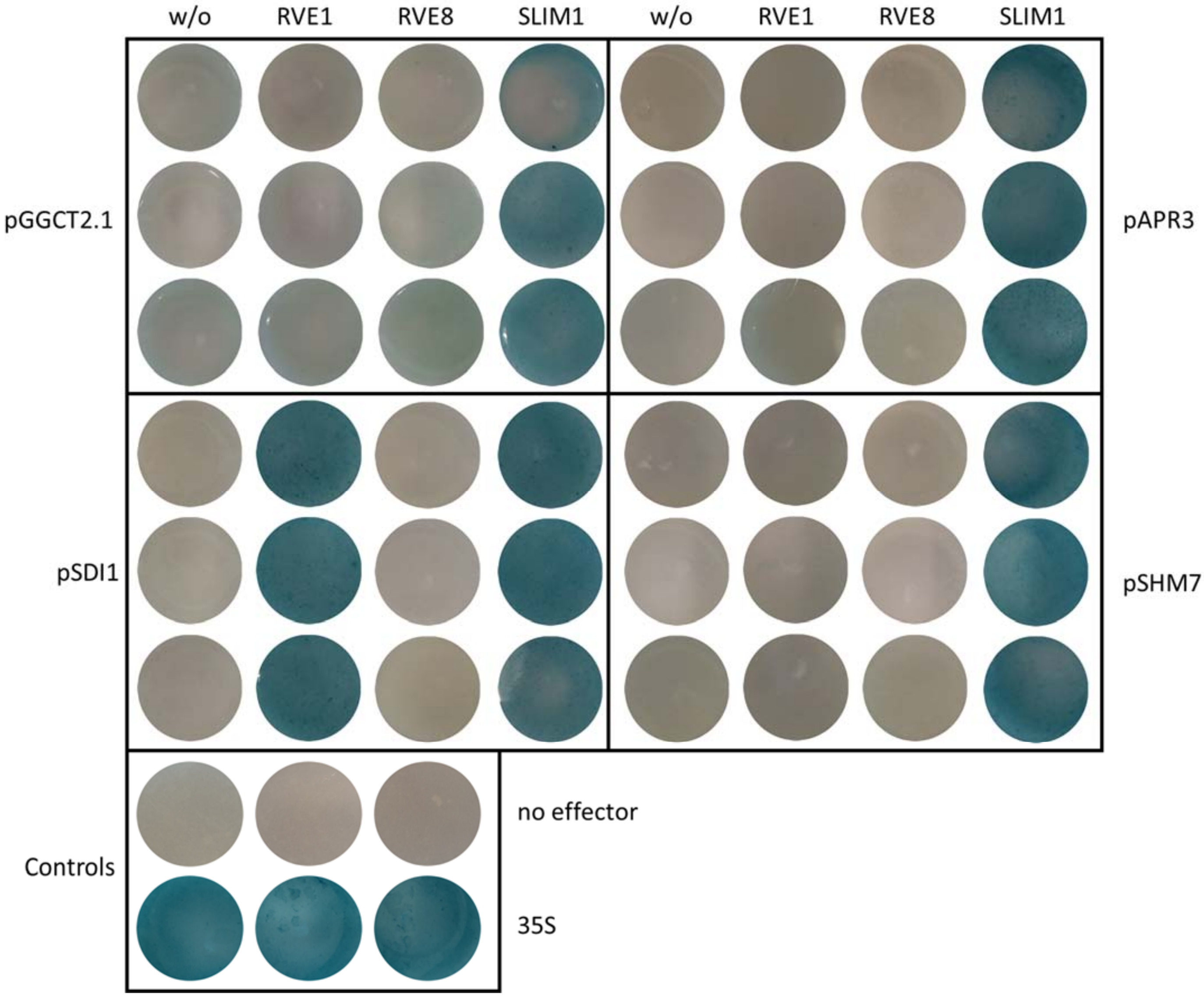

Supplementary Figure S1. Transactivation assays showing interactions of RVE1, RVE8, and SLIM1 with promoters of OAS cluster genes. The promoters of *GGCT2;1, SDI1*, *APR3*, and *SHM7* genes were fused to the *uidA* (GUS) reporter gene. Cultured *A. thaliana* cells were transformed with the supervirulent *Agrobacterium* strain LBA4404.pBBR1MCS. virGN54D containing the reporter constructs and, the effector constructs expressing RVE1, RVE8, and SLIM1 under control of 35S promoter. Histochemical GUS staining of cultured cells was performed 3 d after transformation overnight. 35S::GUS and a promoter construct without effector were used as a positive and negative control, respectively. Shown is a typical well from 3 independent experiments.

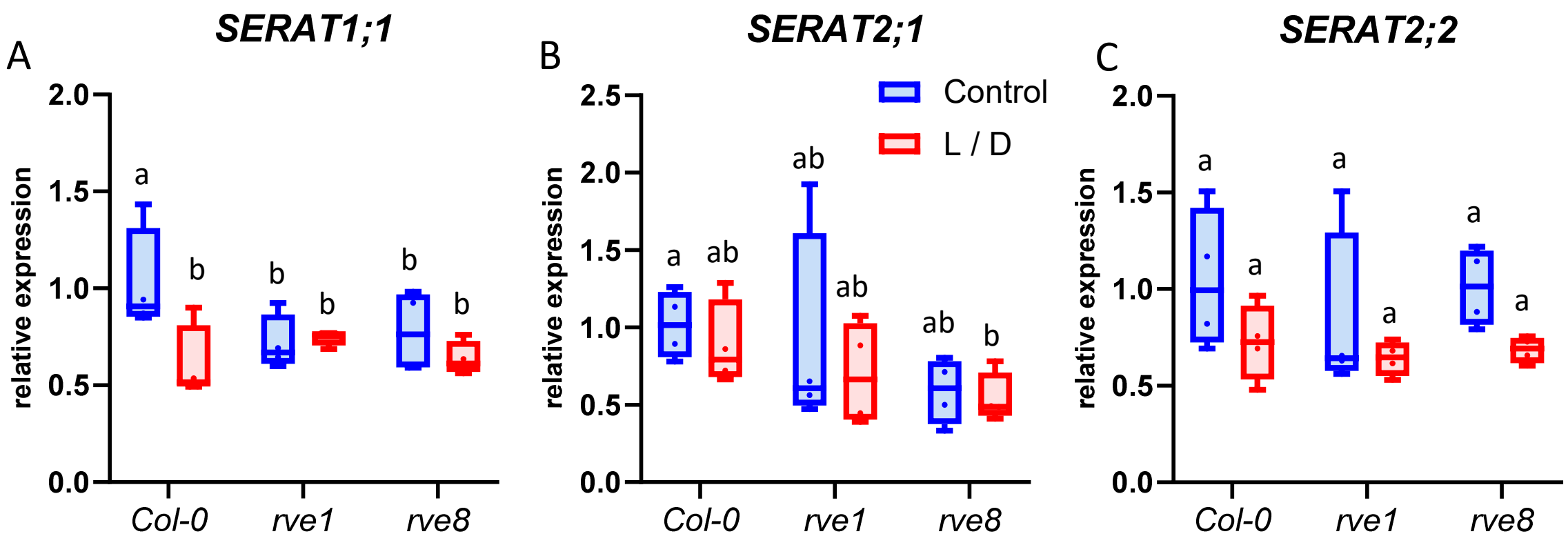

Supplementary Figure S2. Relative expression of the main *SERAT* isoforms after light-dark transition in WT, *rve1* and *rve8*. Arabidopsis *rve1*, and *rve8* mutants and the WT were grown for 17 days on agar plates and transferred to darkness. RNA was isolated from shoots 40 min after the transfer and from control plants grown in light and relative transcript levels of *SERAT1;1* (**A**), *SERAT2;1* (**B**), *SERAT2;2* (**C**) were determined. The RT-qPCR results were obtained from 4 biological and 2 technical replicates, the transcript levels in WT Col-0 control were set to 1. In the boxplots, each dot represents one biological replicate, n = 4; the central line indicates the median; the limits of the box show the upper and lower quartiles; and the whiskers indicate maximum and minimum values. Letters mark values significantly different at $P<0.05$ (Two way ANOVA).

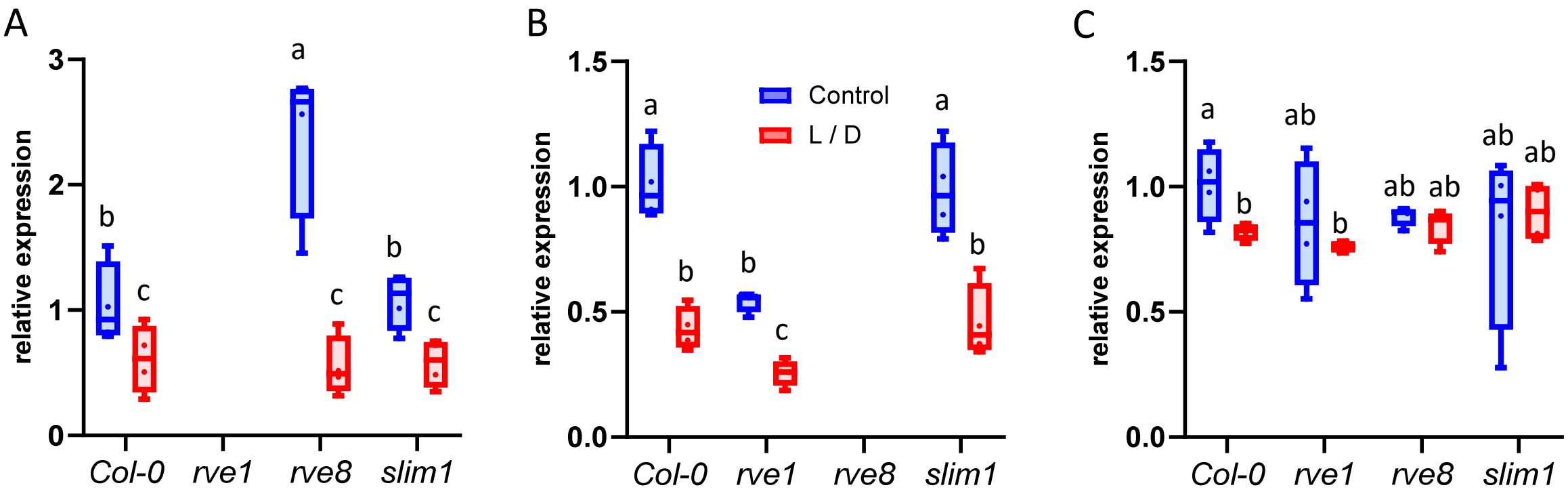

Supplementary Figure S3. Relative expression of *RVE1*, *RVE8* and *SLIM1* after light-dark transition in WT, *slim1-1*, *rve1* and *rve8*. Arabidopsis *rve1*, *rve8*, and *slim1-1* mutants and the WT were grown for 17 days on agar plates and transferred to darkness. RNA was isolated from shoots 40 min after the transfer and from control plants grown in light and relative transcript levels of *RVE1* (**A**), *RVE8* (**B**), *SLIM1* (**C**) were determined. The RT-qPCR results were obtained from 4 biological and 2 technical replicates, the transcript levels in WT Col-0 control were set to 1. In the boxplots, each dot represents one biological replicate, n = 4; the central line indicates the median; the limits of the box show the upper and lower quartiles; and the whiskers indicate maximum and minimum values. Letters mark values significantly different at $P<0.05$ (Two way ANOVA).

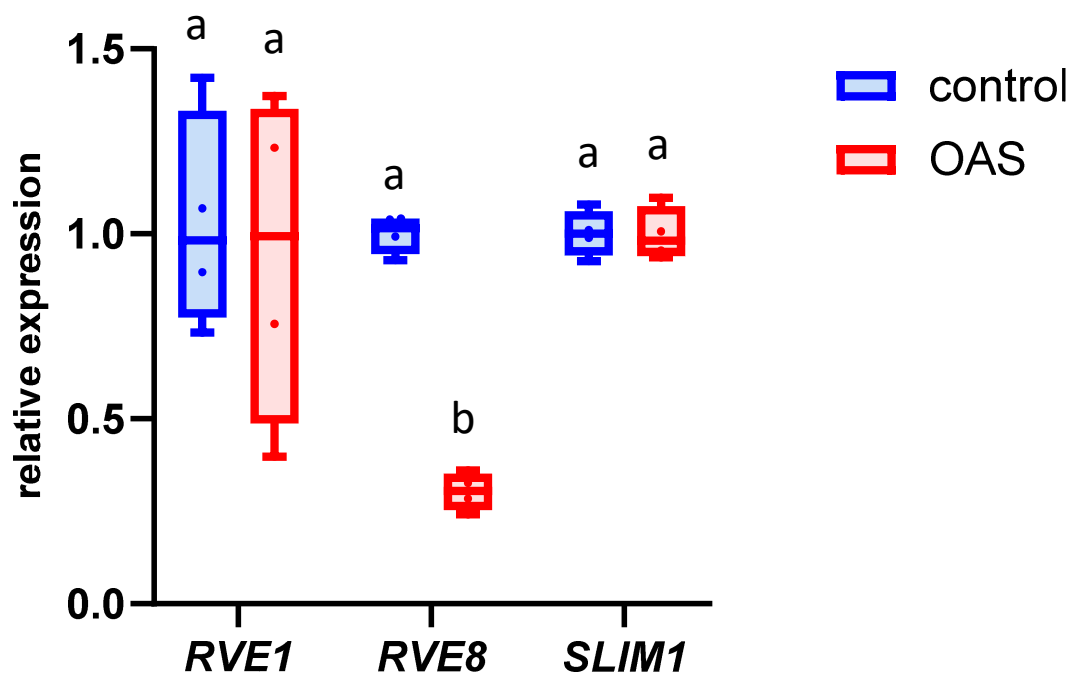

Supplementary Figure S4. Relative expression of *RVE1*, *RVE8* and *SLIM1* after OAS treatment. Arabidopsis WT Col-0 plants were grown for 14 days in hydroculture and treated with 1 mM OAS for 4 h. RNA was isolated from roots and relative transcript levels of *RVE1*, *RVE8*, and *SLIM1* were determined. The RT-qPCR results were obtained from 4 biological and 2 technical replicates, the transcript levels in control were set to 1. In the boxplots, each dot represents one biological replicate, n = 4; the central line indicates the median; the limits of the box show the upper and lower quartiles; and the whiskers indicate maximum and minimum values. Letters mark values significantly different at $P<0.05$ (Two way ANOVA).

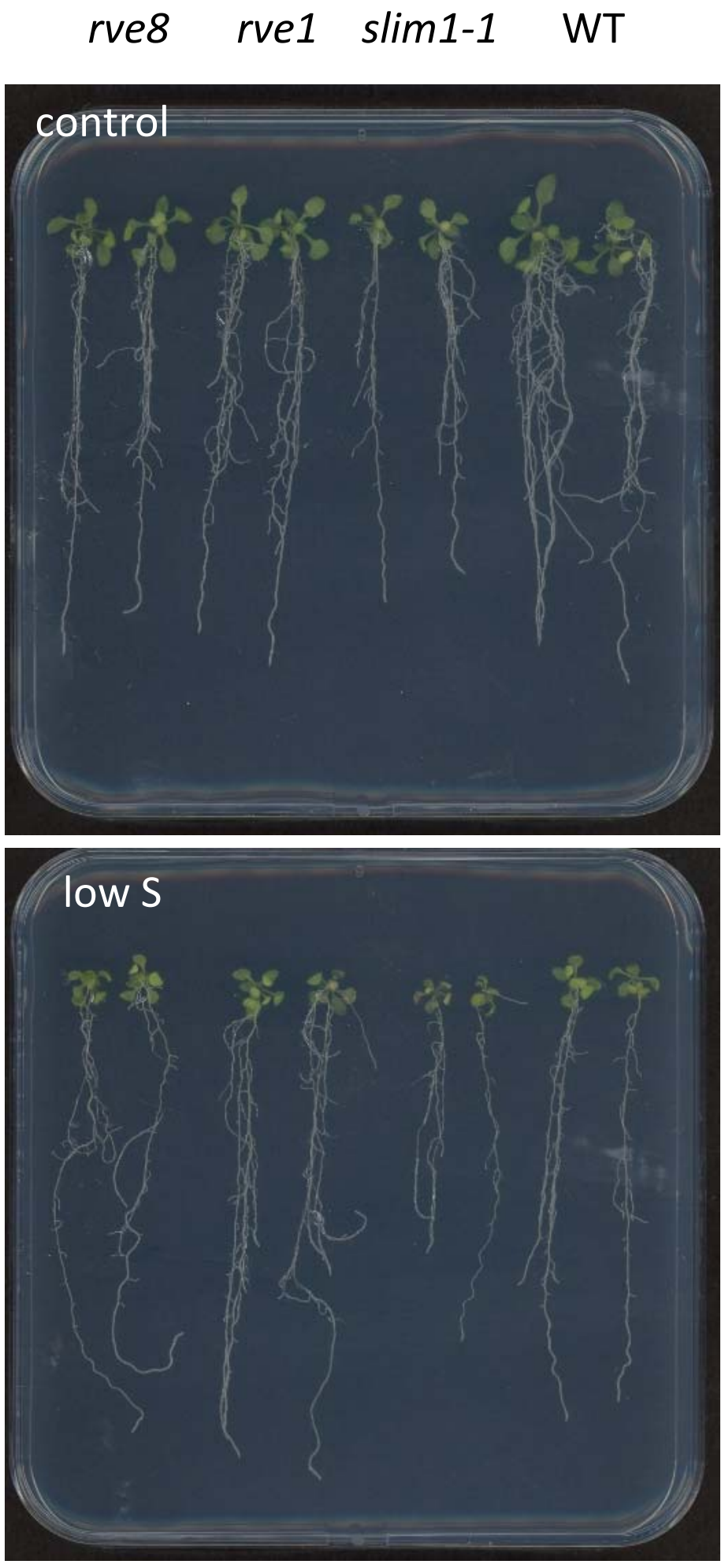

Supplementary Figure S5. Phenotypes of mutants. Arabidopsis WT Col-0, *slim1-1*, *rve1*, and *rve8* plants were grown for 17 days on agarose plates with control (750 µM) or low S (25 µM) sulfate supply.

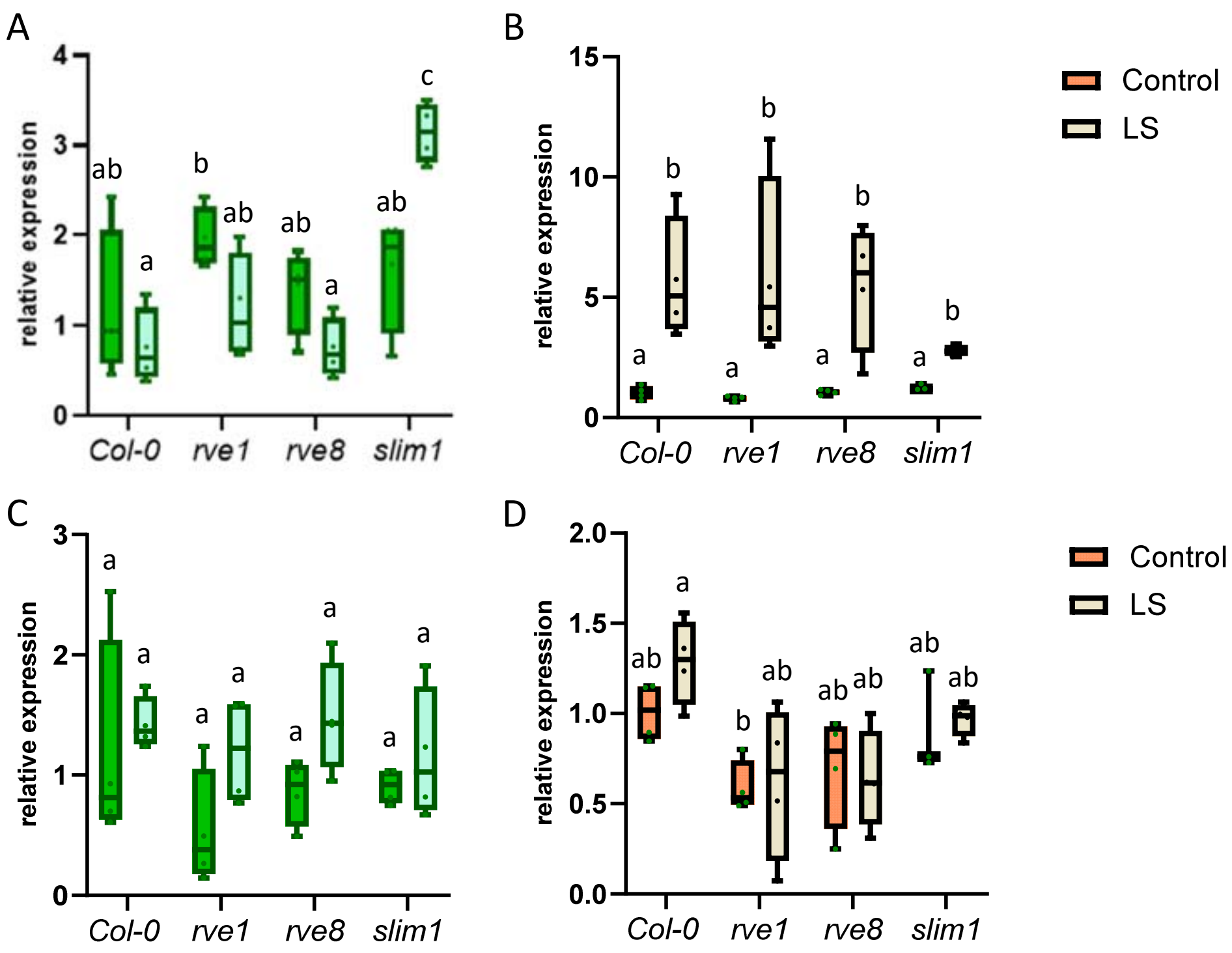

Supplementary Figure S6. Relative expression of *SULTR2;1* and *SiR* in S-deficient plants. Arabidopsis *rve1*, *rve8*, and *slim1* mutants and the WT were grown for 17 days on agarose plates with control sulfate supply (750 µM) or sulfur limitation (LS, 25 µM sulfate). RNA was isolated from roots and relative transcript levels of *SULTR2;1* (A, B) and SiR (C, D) were determined in shoots (A, C) and roots (B, D) by RT-qPCR. The RT-qPCR results were obtained from 4 biological and 2 technical replicates, the transcript levels in WT Col-0 control were set to 1. In the boxplots, each dot represents one biological replicate, n = 4; the central line indicates the median; the limits of the box show the upper and lower quartiles; and the whiskers indicate maximum and minimum values. Letters mark values significantly different at $P<0.05$ (Two-way ANOVA).

Supplementary Table S1. Media composition

Modified Long Ashton

nutrient	final concentration
Ca(NO3)2x4H2O	1.5 mM
KNO3	1 mM
KH2PO4	0.75 mM
MgSO4x7H2O	0.75 mM
Fe-EDTA	1 mM
MnCl2x4H2O	10 µM
H3BO3	50 µM
ZnCl2	1.75 µM
CuCl2	0.5 µM
Na2MoO4	0.8 µM
KI	1 mM
CoCL2x6H2O	0.1 mM
Sucrose	5 g/L
MES hydrate (Sigma-Aldrich)	0.8g/L
Low EEO agarose (Biozym Scientific)	8 g/L

AT-medium

nutrient	final concentration
MS basal salt mixture (Duchefa)	4.4 g/L
Gamborg's vitamin solution (Sigma-Aldrich)	0.5% (v/v)
glucose	30 g/L
2,4-Dichlorophenoxyacetic acid	1 mg/L

Supplementary Table S2. Oligonucleotide primers used in the study.

qPCR

Name	Gene	Sequence (5' →3')
q-TIP41-F	AT4G34270	GAACTGGCTGACAATGGAGTG
q-TIP41-R		ATCAACTCTCAGCCAAAATCG
q-SDI1-F	AT5G48850	TCCCTGTGGAGACACTCCTT
q-SDI1-R		CCATCTCCGGGTTCTTCTCT
q-SDI2-F	AT1G04770	GTCCTTATGTCCGAGCGAAG
q-SDI2-R		TCTCGCTTTAATCGCTATCCA
q-LSU1-F	AT3G49580	TTAAGTTGTGGCAGCGAACG
q-LSU1-R		CCATGAGGAAGAGCATGCGA
q-APR3-F	AT4G21990	CCAATCAAGTATCCATCAGAGAAG
q-APR3-R		CCGAACAAGATTCAAGAAAGATG
q-GGCT2;1-F	AT5G26220	TCCACCGGAGCTATTTGC
q-GGCT2;1-R		CGTTCCAAGTACTCCATTGCT
q-SHM7-F	AT1G36370	TCTTAGAAGAAAATGCAGACTGG
q-SHM7-R		CACACACTTTCTCGTAGACTTTCC
q-SULTR1.2-F	AT1G78000	ATCCGTTTTCAAAGCAGCTC
q-SULTR1.2-R		TCAAGAATGATGCACCAATGA
q-SLIM1-F	AT1G73730	TGACTTGGACTTTGACTATGGTG
q-SLIM1-R		CTATTGCCATGTCCTTTTGAACT
q-RVE1-F	AT5G17300	CTCTTTGGGAAGACAGTTTTGG
q-RVE1-R		GATGATCCTTCGTTTTGGACAG
q-RVE8-F	AT3G09600	GGGAAGCTCAAGCCGAACAGTATC
q-RVE8-R		GGCCTCTCGTTTCAGGATCAAAG

Cloning

Name	Gene	Sequence (5' →3')
cRVE8-F	AT3G09600	CACCATGAGCTCGTCGCC
cRVE8-R		TTATGCTGATTTGTCGCTTGTTGAG
cRVE1-F	AT5G17300	CACCATGGCGTCGTCTCC
cRVE1-R		TTATAAGTGGAGATGAATCTCATGC
cSLIM1-F	AT1G73730	CACCATGGGCGATCTTGC
cSLIM1-R		CTAAGCTCCAAACCATGAGAAATC
proGGCT2;1-F	AT5G26220	CACCAATTGAATCAGCTAACTTAAT
proGGCT2;1-R		CTTTGATCCTTAGCCTCACAC
proSDI1_2kb-I	AT5G48850	CACCTTCTCTTTGTCTCTCT
proSDI1_2kb-R		CTTTTTTTCCTCTGTTTTTCTCTTT
M13-F	pENTRY-TOPO ligation	GTAAAACGACGGCCAG
M13-R		CAGGAAACAGCTATGAC
B1	pGWB2/3 ligation	ACAAGTTTGTACAAAAAAGCAGGCT
B2		ACCACTTTGTACAAGAAAGCTGGGT
